# Integration Analysis of *JAK2* or *RUNX1* Mutation With Bone Marrow Blast Can Improve Risk Stratification in the Patients With Lower Risk Myelodysplastic Syndrome

**DOI:** 10.3389/fonc.2020.610525

**Published:** 2021-01-13

**Authors:** Ying Fang, Juan Guo, Dong Wu, Ling-Yun Wu, Lu-Xi Song, Zheng Zhang, You-Shan Zhao, Chun-Kang Chang

**Affiliations:** Department of Hematology, Shanghai Jiao Tong University Affiliated Sixth People’s Hospital, Shanghai, China

**Keywords:** lower-risk myelodysplastic syndrome, revised International Prognostic Scoring System, next-generation sequencing, bone marrow blast, risk factor

## Abstract

Despite the improvements in prognostication of the revised International Prognostic Scoring System (IPSS-R) in myelodysplastic syndrome (MDS), there remain a portion of patients with lower risk (low/intermediate risk, LR) but poor prognostics. This study aimed to evaluate the relative contribution of mutational status when added to the IPSS-R, for estimating overall survival (OS) and progression-free survival (PFS) in patients with LR-MDS. We retrospectively analyzed clinical and laboratory variables of 328 patients diagnosed with MDS according to the FAB criteria. Twenty-nine-gene NGS assay was applied to bone marrow samples obtained at diagnosis. 233 (71.04%) patients were classified as LR-MDS. Univariate analysis showed association between inferior outcome (OS and PFS) and presence of *JAK2* (p = 0.0177, p = 0.0002), *RUNX1* (p = 0.0250, p = 0.0387), and *U2AF1* (p = 0.0227, p = 0.7995) mutations. Multivariable survival analysis revealed *JAK2* (p < 0.0001) and *RUNX1* (p = 0.0215) mutations were independently prognostic for PFS in LR-MDS. Interestingly, bone marrow blast >1.5% could further predict disease progression of patients with LR-MDS (HR 8.06, 95%CI 2.95–22.04, p < 0.0001). Incorporation of *JAK2*, *RUNX1* mutation and bone marrow blast in the IPSS-R can improve risk stratification in patients with LR-MDS. In summary, our result provided new risk factors for LR-MDS prognostics to identify candidates for early therapeutic intervention.

## Introduction

Myelodysplastic syndrome (MDS) represents a heterogeneous group of clonal hematopoietic disorders with diverse clinical manifestations such as ineffective bone marrow (BM) hematopoiesis, peripheral blood cytopenia and variable propensity to progress to acute myeloid leukemia (AML) (1).

30% of MDS patients may progress to acute myeloid leukemia (2, 3). This emphasizes the need to stratify MDS patients into low or high risk for progression to guide optimal MDS treatment. Currently, revised International Prognostic Scoring System (IPSS-R) was regarded as the dominant prognostic scoring system which is based on a comprehensive cytogenetic scoring system (MDS cytogenetic scoring system) and defines 5 risk groups with different clinical outcomes: very low risk, low risk, intermediated risk, high risk, and very high risk (4). Patients with lower-risk (low/intermediate risk according to IPSS-R) myelodysplastic syndrome (LR-MDS) account for approximately two-thirds of patients with MDS. There remains a proportion of patients with lower risk but poor prognosis (5). Meanwhile, with the advance of next generation sequencing (NGS), a number of driver mutations were found in MDS (6) with diverse prognosis under the standard immunochemotherapy (7, 8).

In this study, we analyzed the genetic and clinical characteristics of a large cohort of patients with LR-MDS according to IPSS-R, and identify new risk factors which could further segregate patients into different risk groups, thus, to provide rationale to refine risk stratification and improve the accuracy of current prognostic scoring system.

## Materials and Methods

### Patients and Clinical Methods

A total of 328 MDS patients analyzed in this study (**Table 1**). 233 (71.04%) patients were classified as LR-MDS (**Table 2**). Enrolment occurred between April 2016 and September 2019, and the median observation duration was approximately 19 months (range 0.3–168.0). All samples were obtained from patients treated at the Department of Hematology, Shanghai Jiao Tong University Affiliated Sixth People’s Hospital. Informed consent was obtained from all subjects according to institutional review board-approved protocols, which were carried out in accordance with the Declaration of Helsinki. All experiments were approved by the Ethics Committee of Shanghai Jiao Tong University Affiliated Sixth People’s Hospital. All methods used in this study were performed in accordance with approved guidelines. Response to treatment was assessed using the International Working Group (IWG) Response Criteria, which were revised in 2006 (9).

**Table 1:** Clinical characteristics of patients with MDS.

Characteristics	Enrolled patients (n = 328)
**Age**	
≤60	162 (49.39%)
>60	166 (50.61%)
**Gender**	
Male	204 (62.20%)
Female	124 (37.80%)
**Diagnosis**	
REAB-I/II	70 (21.34%)
5q-	3 (0.91%)
MDS/MPN	2 (0.61%)
RCUD	40 (12.20%)
RARS	29 (8.84%)
RCMD	184 (56.10%)
**Cytogenetics**	
Normal	220 (67.07%)
Complex	10 (3.05%)
-7/del(7q)	16 (4.88%)
Others	82 (25.00%)
**BM blast%**	
0–2%	198 (60.37%)
>2–<5%	53 (16.16%)
5–10%	47 (14.33%)
>10%	21 (6.40%)
NA	9 (2.74%)
**Absolute Neutrophil Count^†^**	
≥0.8	208 (63.41%)
<0.8	120 (36.59%)
**Hemoglobin, g/dL**	
≥10	67 (20.43%)
8–<10	60 (18.29%)
≥10	201 (61.28%)
**PLT^†^**	
≥100	104 (31.71%)
50–<100	72 (21.95%)
<50	152 (46.34%)
**MF**	
No	283 (86.28%)
Yes	35 (10.67%)
NA	10 (3.05%)

^†^×10^9/L.

**Table 2:** Clinical characteristics of patients with LR-MDS (n = 233).

Characteristics	LR-MDS patients (n = 233)
**Age**	
≤60	132 (56.65%)
>60	101 (43.35%)
**Gender**	
Male	141 (60.52%)
Female	92 (39.48%)
**Diagnosis**	
REAB-I/II	19 (8.15%)
5q-	2 (0.86%)
RCUD	31 (13.30%)
RARS	28 (12.02%)
RCMD	152 (65.24%)
CMML	1 (0.43%)
**Cytogenetics**	
Normal	182 (78.11%)
Complex	1 (0.43%)
-7/del(7q) Others	3 (1.29%) 47 (20.17%)
**BM blast%**	
0–2%	175 (75.11%)
>2–<5%	35 (15.02%)
5–10%	15 (6.44%)
>10%	2 (0.86%)
NA	6 (2.58%)
**Absolute Neutrophil Count^†^**	
≥0.8	154 (66.09%)
<0.8	79 (33.91%)
**Hemoglobin, g/dL**	
≥10	47 (20.17%)
8–<10	41 (17.60%)
<8 **PLT^†^**	145 (62.23%)
≥100	86 (36.91%)
50–<100	48 (20.60%)
<50	99 (42.49%)
**MF**	
No	209 (89.70%)
Yes	17 (7.30%)
NA	7 (3.00%)

^†^×10^9/L.

All patients with LR-MDS received disease‐modifying therapeutic strategies according to NCCN Guidelines (10), which included supportive care, such as red blood cell (RBC) transfusions, erythroid stimulating agents (ESAs), granulocyte-macrophage colony stimulating factors (GM-CSF), and lenalidomide. Patients especially those with hypocellular BM receive immunosuppressive therapy and iron chelation is indicated in patients with iron overload. More aggressive therapies such as hypomethylating agents are usually not administered to patients with LR-MDS. Bone marrow (BM) samples were harvested from patients (n = 328) at diagnosis before treatment. The percentage of BM blasts was assessed from total nucleated cells (TNCs). All samples were kept frozen at -80°C until mutational analysis performed.

### Mutational Analysis

The mutational analysis by next‐generation sequencing (NGS) was carried out for all patients whose DNA was of adequate quality, following procedures as previously described. Twenty-nine-gene panel were listed in **Table S1**. All assays were performed blinded to the study end points, by pathologists who were not involved in patient management. Mutational data underlying the study is available in **Table S2**.

### Statistical Analysis

All statistical analyses were conducted with SPSS software, version 23.0 (SPSS, Inc., Chicago, IL, USA). Progression-free survival (PFS) was measured from the date when diagnosed to the date when disease progression was recognized or the date of last follow-up. Overall survival (OS) was measured from the date when diagnosed to the date of death or last follow-up. Distributions of PFS and OS were estimated by the method of Kaplan-Meier, and compared between subgroups using the log rank test. Univariate-, multivariate hazard estimates were generated with Cox proportional hazards models. Categorical variables were compared using Fisher’s exact test or χ^2^ test as appropriate. A two-sided p value of <0.0500 was considered statistically significant.

## Results

### Patient Characteristics and Overall Predicted Outcome of International Prognostic Scoring System

**Figure 1:**
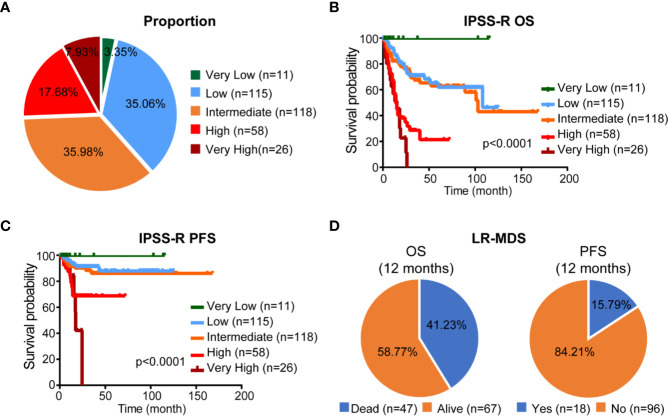
Proportion and prognosis of patients according to IPSS-R classification. **(A)** Proportion of patients according to IPSS-R classification. **(B, C)** Kaplan-Meier curves for OS and PFS according to IPSS-R classification. **(D)** Proportion of different clinical outcome of LR-MDS.

A total of 328 *de novo* MDS patients, diagnosed according to the 2008 World Health Organization (WHO) classification, were included in our study. Their IPSS-R score was calculated based on their initial presentation to our institution. Among the total cohort, 11 patients (3.35%) had very low-risk disease, 115 patients (35.06%) had low-risk disease, 118 patients (35.98%) had intermediate-risk disease, 58 patients (17.68%) had high-risk disease and 26 patients (7.93%) had very high risk (**Figure 1A**). OS and PFS were shown in **Figures 1B, C**. Median OS was 19 months [95% confidence interval (CI) 26.2–33.0], PFS median follow-up was 18 months (range 0.3–168.0). However, as shown in **Figure 1D**, IPSS-R still has limitation to identify patients with lower risk but poor prognosis: 47/114 (41.23%) patients with LR-MDS were dead within 12 months and 18/114 (15.79%) patients had disease progression within 12 months.

### Mutational Analysis

**Figure 2:**
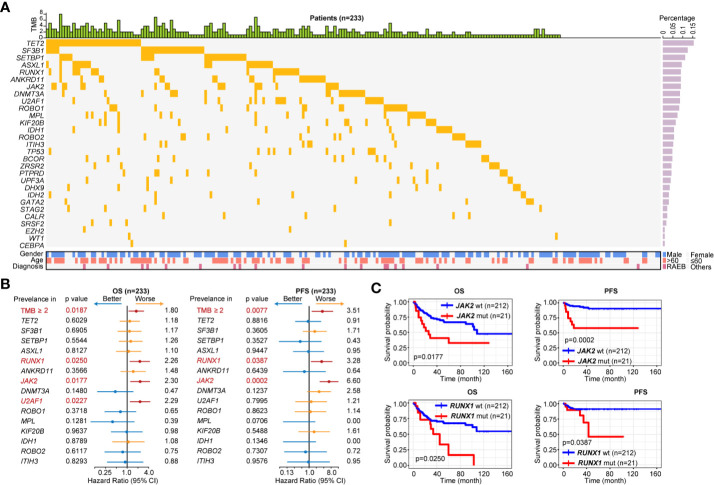
Gene mutation pattern of patients with LR-MDS. **(A)** Gene mutation pattern of patients with LR-MDS (n = 233). **(B)** Overall survival (OS) and progression-free survival (PFS) analysis of highly recurrent mutated genes among patients with LR-MDS (n = 233). **(C)** Kaplan-Meier curves for OS and PFS according to mutation status of *JAK2* (top) and *RUNX1* (bottom).

We then focused on patients with LR-MDS (n = 233) based on IPSS-R, and investigated their genetic characteristics using 29-gene NGS assay (**Figure 2A**). One hundred ninety-five (83.69%) patients contained ≥1 mutation [median (IQR): 2 (1–3)]. Fifteen genes were recurrent mutated (mutation rate > 5%). The most frequently mutated gene was *TET2* (15.45%) followed by *SF3B1* (12.45%), *SETBP1* (11.16%), *ASXL1* (9.44%), *RUNX1* (9.01%), *ANKRD11* (9.01%), *JAK2* (9.01%), *DNMT3A* (8.58%), *U2AF1* (8.58%), *ROBO1* (8.15%), *MPL* (7.30%), *KIF20B* (6.44%), *IDH1* (5.58%), *ROBO2* (5.58%), and *ITIH3* (5.15%). We further investigate the prognostic influence of tumor mutation burden (TMB, ≥2 mutations) and recurrent mutations, univariate analysis indicated that TMB ≥2, *RUNX1* and *JAK2* mutations were associated with inferior OS and PFS, and *U2AF1* mutations was associated with inferior OS [hazard ratio (HR) > 1.5, p < 0.0500, **Figure 2B**]. Kaplan-Meier curves of PFS and OS according to mutation status of *RUNX1* and *JAK2* were shown in **Figure 2C**. Multivariable survival analysis revealed *JAK2* [HR 6.58, 95%CI 2.72–15.91, p < 0.0001] and *RUNX1* [HR 3.25, 95%CI 1.19–8.89, p = 0.0215] mutations were independently prognostic for PFS in LR-MDS.

### Clinical and Laboratory Characteristics

To further investigate the clinical implications of *JAK2* and *RUNX1* mutation, clinical and laboratory data were analyzed including age, gender, diagnosis, cytogenetics, percentage of bone marrow (BM) blast, absolute neutrophil count (ANC), hemoglobin (Hb), PLT and myelofibrosis (MF) status with Gomori staining positive (++~++++). As shown in **Table 3**, patients were reclassified according to their mutation status. *JAK2*-mutated patients had lower ANC (<0.8×10^^^9/L, 76.19 vs. 29.72%, p < 0.0001) than *JAK2* wildtype patients and tended to be more responsible for MF (19.05 vs. 6.13%, p = 0.0129). Elderly patients (>60 y) tended to have recurrent *RUNX1* mutation (p = 0.0098), and *RUNX1-*mutated patients had higher percentage of BM blast (>2%) when compared to those with *RUNX1* wildtype (47.62 vs. 19.81%, p = 0.0452). We then evaluated the prognostic influence of BM blast (%) from 237 available LR-MDS patients, and tried to identify a suitable cutoff to improve PFS prediction.

**Table 3:** Clinical characteristics of patients with LR-MDS (n = 233).

Characteristics	*JAK2* mut (n = 21)	*JAK2* wt (n = 212)	p value	*RUNX1* mut (n = 21)	*RUNX1* wt (n = 212)	p value
**Age**			0.8181			**0.0098**
≤60	11 (52.38%)	121 (57.08%)		6 (28.57%)	126 (59.43%)	
>60	10 (47.62%)	91 (42.92%)		15 (71.43%)	86 (40.57%)	
**Gender**			0.6436			0.6436
Male	14 (66.67%)	127 (59.91%)		14 (66.67%)	127 (59.91%)	
Female	7 (33.33%)	85 (40.09%)		7 (33.33%)	85 (40.09%)	
**Diagnosis**			0.3360			0.7973
REAB-I/II	1 (4.76%)	18 (8.49%)		3 (14.29%)	16 (7.55%)	
5q-	1 (4.76%)	1 (0.47%)		0 (0.00%)	2 (0.94%)	
RCUD	1 (4.76%)	30 (14.15%)		2 (9.52%)	29 (13.68%)	
RARS	3 (14.29%)	25 (11.79%)		2 (9.52%)	26 (12.26%)	
RCMD	15 (71.43%)	137 (64.62%)		14 (66.67%)	138 (65.09%)	
CMML	0 (0.00%)	1 (0.47%)		0 (0.00%)	1 (0.47%)	
**Cytogenetics**			0.0803			0.4220
Normal	14 (66.67%)	168 (96.00%)		14 (66.67%)	168 (79.25%)	
Complex	1 (4.76%)	0 (0.00%)		0 (0.00%)	1 (0.47%)	
-7/del(7q) Others	0 (0.00%) 6 (28.57%)	3 (1.71%) 41 (2.29%)		0 (0.00%) 7 (33.33%)	3 (1.42%) 40 (18.87%)	
**BM blast%**			0.3369			**0.0452**
0–2%	15 (71.43%)	160 (75.47%)		11 (52.38%)	164 (77.36%)	
>2–<5%	3 (14.29%)	32 (15.09%)		7 (33.33%)	28 (13.21%)	
5–10%	1 (4.76%)	14 (6.60%)		3 (14.29%)	12 (5.66%)	
>10%	0 (0.00%)	2 (0.94%)		0 (0.00%)	2 (0.94%)	
NA	2 (9.52%)	4 (1.89%)		0 (0.00%)	6 (2.83%)	
**Absolute Neutrophil Count^†^**			**<0.0001**			0.1367
≥0.8	5 (23.81%)	149 (70.28%)		18 (85.71%)	147 (69.34%)	
<0.8	16 (76.19%)	63 (29.72%)		3 (14.29%)	65 (30.66%)	
**Hemoglobin, g/dL**			0.8490			0.0648
≥10	3 (14.29%)	44 (20.75%)		8 (38.10%)	39 (18.40%)	
8–<10	4 (19.05%)	37 (17.45%)		1 (4.76%)	40 (18.87%)	
<8 **PLT^†^**	14 (66.67%)	131 (61.79%)	0.3528	12 (57.14%)	133 (62.73%)	0.3056
≥100	9 (42.86%)	77 (36.32%)		6 (28.57%)	80 (37.74%)	
50–<100	6 (28.57%)	42 (19.81%)		7 (33.33%)	41 (19.34%)	
<50	6 (28.57%)	93 (43.87%)		8 (38.10%)	91 (42.92%)	
**MF**			**0.0129**			0.8275
No	15 (71.43%)	194 (91.51%)		19 (90.48%)	190 (89.62%)	
Yes	4 (19.05%)	13 (6.13%)		2 (9.52%)	15 (7.08%)	
NA	2 (9.52%)	5 (2.36%)		0 (0.00%)	7 (3.30%)	

^†^×10^9/L. All the p value in bold were <0.05, which were considered to be statistically significant.

**Figure 3:**
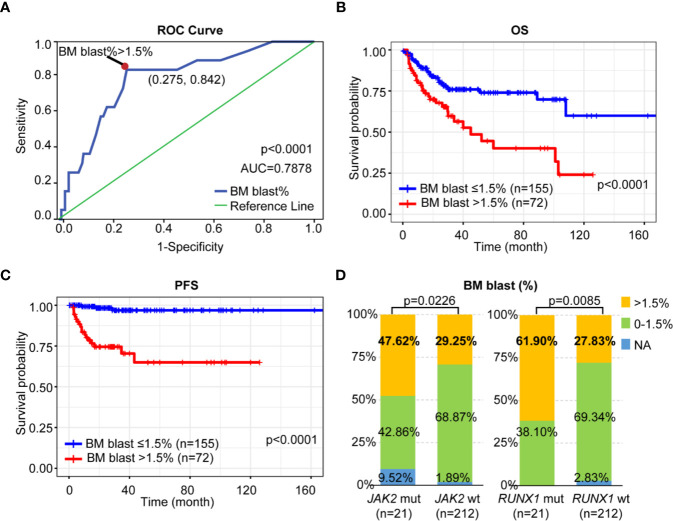
Prognostic impact of BM blast on LR-MDS. **(A)** ROC curve of BM blast-based model. **(B)** Kaplan-Meier curves for OS according to the percentage of BM blast. **(C)** Kaplan-Meier curves for PFS according to the percentage of BM blast. **(D)** Distribution of BM blast (%) among LR-MDS patients with or without *JAK2* or *RUNX1* mutations.

Subsequently, we classified patients according to their PFS status (progression or not) and conducted receiver operating curve (ROC) analyses to assess how the BM blast (%) could behave in predicting prognosis (**Figure 3A**). After detection, it was found that the area under the BM blast (%) curve was 0.7878 and the 95% CI was 0.6857–0.8900. When the cutoff value was 1.50%, the optimal specificity was 72.46%, the sensitivity 84.21% and the Youden index 0.5667 which revealed that at the cutoff (BM blast > 1.5%) could further improve risk stratification in patients with LR-MDS. As shown in **Figures 3B, C**, LR-MDS patients with BM blast >1.5% has an inferior OS and PFS when compared with those with BM blast ≤1.5% (median OS 20.33 months, 95%CI 14.00–27.00 vs. median OS 26.00 months, 95%CI 23.00–30.43, p < 0.0001; median PFS 17.50 months, 95%CI 12.47–27.00 vs. median PFS 26.00 months, 95%CI 23.00–30.43, p < 0.0001).

Interestingly, we found both *JAK2* and *RUNX1-*mutated patients had high percentage of BM blast (>1.5%) as when compared to those with wildtype respectively (47.62 vs. 29.25%, p = 0.0226; 61.90 vs. 27.83%, p = 0.0085) (**Figure 3D**).

### Prediction of Prognosis of Low/Intermediate Risk-Myelodysplastic Syndrome

**Figure 4:**
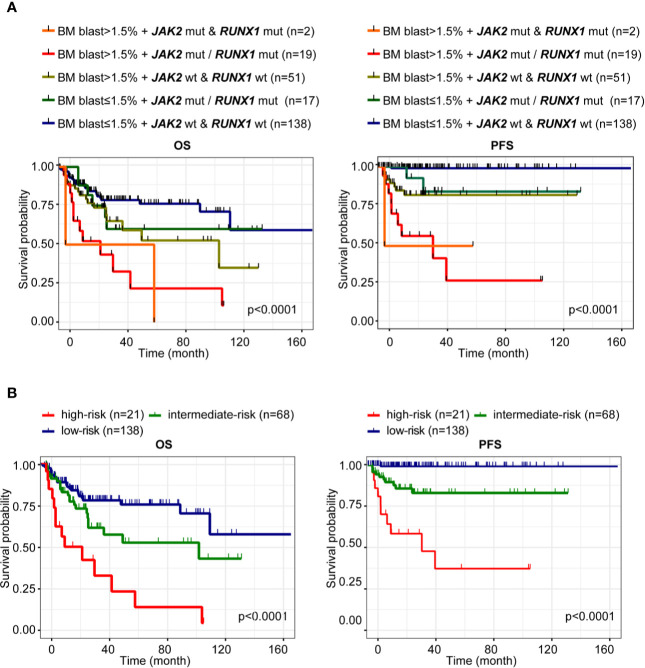
Prediction of prognosis of LR-MDS. **(A)** Kaplan-Meier curves for OS and PFS according to BM blast (%) and mutation status of *JAK2* and *RUNX1* of patients with LR-MDS. **(B)** Kaplan-Meier curves for OS and PFS of the following three subgroups: low-risk subgroup (BM blast ≤1.5% without *JAK2* and *RUNX1* mutation), intermediate-risk subgroup (BM blast ≤1.5% with *JAK2* or *RUNX1* mutation, BM blast >1.5% without *JAK2* and *RUNX1* mutation), and high-risk subgroup (BM blast >1.5% with *JAK2* and/or *RUNX1* mutation).

According to BM blast (%) and mutation status of *JAK2* and *RUNX1*, patients with LR-MDS could be further stratified into following three subgroups: low-risk subgroup (BM blast ≤1.5% without *JAK2* and *RUNX1* mutation), intermediate-risk subgroup (BM blast ≤1.5% with *JAK2* or *RUNX1* mutation, BM blast >1.5% without *JAK2* and *RUNX1* mutation), and high-risk subgroup (BM blast >1.5% with *JAK2* and/or *RUNX1* mutation) (**Figure 4A**). Finally, when incorporated *JAK2*, *RUNX1* mutation and bone marrow blast, we further segregate patients with LR-MDS into following three risk groups: low-risk subgroup (60.79%, 138 of 227 patients) with median OS of 25.93 months (95%CI 23.00–30.33) and median PFS 25.93 months (95%CI 23.00–30.33); intermediate-risk subgroup (29.96%, 68 of 227 patients) with median OS of 24.00 months [95%CI 18.00–29.63, HR 1.73 (0.96–3.12), p = 0.0495] and median PFS 24.00 months [95%CI 17.00–29.63, HR 20.16 (5.75–70.72), p < 0.0001] and high-risk subgroup (9.25%, 21 of 227 patients) with median OS of 13.00 months [95%CI 7.00–33.07, HR 4.50 (1.65–12.29), p < 0.0001] and median PFS 12.47 months [95%CI 6.60–33.07, HR 81.89 (12.28–546.20), p < 0.0001] as shown in **Figure 4B**.

## Discussion

MDS is a heterogeneous disease with various clinicopathological and molecular features (11, 12). Thus, accurate prognostication and risk stratification is important for patients with MDS to further guide risk-adapted therapy (13). IPSS (14), IPSS-R, MD Anderson Risk Model Score (MDAS) (15), WHO-classification based Prognostic Scoring System (WPSS) (16) and refined WPSS (WPSS-R) (17) are the most widely adopted models in clinical practice for risk stratification of MDS in the latest years. IPSS-R has been recognized as the score with the best prognostic capability in MDS (18–21). However, some concerns still exist about the real prognostic significance of the lower risk category, patients classified into intermediate risk group showed an outcome closer to the expected in higher risk MDS patients (22). Patients with lower risk still carry a significant excess mortality (23).

We investigated genetic characteristics of patients with LR-MDS using NGS assay and examined the prognostic utility of 15 recurrent mutated genes (mutation rate > 5%). We found *RUNX1* (9.01%) and *JAK2* (9.01%) mutations were associated with inferior OS and PFS among patients with LR-MDS. *RUNX1*, a member of the core-binding factor family of transcription factors, is indispensable for the establishment of definitive hematopoiesis (24). *RUNX1* is one of the most frequently mutated genes in MDS, accounting for roughly 10% of the cases as previous reported (25, 26). It has been reported that MDS patients with *RUNX1* mutations had a higher risk and shorter latency for progression to AML (27). However, the exact role of *RUNX1* in progression of MDS to AML is not known. In our study, among patients with LR-MDS, cytogenetic changes such as deletion of the entire chromosome 7 or its long arm (-7/7q-) was not common in mutated *RUNX1* patients. It’s worth noting that patients with *RUNX1* mutation tended to had higher percentage of BM blast (> 2%) when compared to those without *RUNX1* mutation which further confirmed the pathogenic role of *RUNX1* mutation in hematopoietic defects and propensity to leukemogenesis.

Recently, *JAK2* mutations have been reported to not only be associated with inferior survival in MDS receiving hematopoietic stem cell transplantation (HSCT) (28), but also in LR-MDS (29). *JAK2* mutation leads to constitutive activation of the JAK2/STAT3 pathway which further resulted in growth factor independence, increased proliferation, and differentiation failure (30). *JAK2* mutations are mainly found in patients with myeloproliferative characteristics (31). It has been reported that *JAK2* mutation patients had significantly higher hemoglobin levels, white blood cell and platelet counts compared to unmutated patients (32, 33). In our study, we found *JAK2*-mutated patients had lower ANC (<0.8×10^9/L, 76.19 vs. 29.72%, p < 0.0001) than *JAK2* wildtype patients and tended to be more responsible for MF (19.05% vs. 6.13%, p=0.0129) which further confirmed *JAK2* mutations reinforced the pathogenesis of MDS, may be involved in disease progression in LR-MDS.

TP53 has also had a poor prognosis in LR-MDS with a relative low mutation rate (4.72%) than frequent *RUNX1* or *JAK2* mutation (9.01%). Kaplan-Meier curves for OS and PFS according to *TP53* status were shown in **Supplementary Figure S1**. However, other mutations which are known to confer poor prognosis including *ASXL1* and *SRSF2* were not shown to affect prognosis in our cohort. Mutation of *ASXL1* gene has been reported to be a molecular marker of disease progression in MDS, in particular its high frequency in REAB-I/II (31.00%) (34). However, only 19 patients with REAB-I/II were included in our cohort with a relatively low *ASXL1* mutation rate 5.26% in REAB-I/II. The mutation rate of *SRSF2* were only 1.72% in our cohort of LR-MDS. Accordingly, *ASXL1* and *SRSF2* mutation did not show significant correlation with the prognosis in our cohort may due to the different proportion of disease classification of enrolled patients.

Proportion of BM blasts has been demonstrated as an independent prognostic factor in MDS. It has been reported that reconsidering the methods of enumerating bone marrow blasts could improve outcome prediction (35). In our study, we found both *JAK2* and *RUNX1-*mutated patients had high percentage of BM blast (> 1.5%), BM blast (> 1.5%) adequately predicted OS and PFS respectively (p < 0.0001, p < 0.0001) among LR-MDS patients.

In conclusion, integrative mutational and clinical analyses reveal that considering *JAK2, RUNX1* mutation status and BM blast percentage (> 1.5%) can further improve risk stratification of disease progression and over survival among the patients with LR-MDS in the context of IPSS-R.

## Data Availability Statement

The mutational data underlying the study is available in **Table S2**.

## Ethics Statement

The studies involving human participants were reviewed and approved by the Ethics Committee of Shanghai Jiao Tong University Affiliated Sixth People’s Hospital. The patients/participants provided their written informed consent to participate in this study.

## Author Contributions

C-KC and Y-SZ contributed to the conception and design of the study; DW, L-YW, L-XS and ZZ recruited the patients and collected the data; YF was responsible for the clinical and mutational analysis; JG assisted with biological sample preparation; YF and Y-SZ wrote the manuscript. C-KC and Y-SZ supervised the study. All authors contributed to the article and approved the submitted version.

## Funding

This study was supported by research funding from the National Natural Science Foundation of China (81770121).

## Conflict of Interest

The authors declare that the research was conducted in the absence of any commercial or financial relationships that could be construed as a potential conflict of interest.

## Supplementary Material

Supplementary Table 1 Genes for targeted sequencing.

Supplementary Table 2 Somatic mutations of hypermutated genes in LR-MDS.

Supplementary Figure 1 Kaplan-Meier curves for OS and PFS according to TP53 status.
