# Supplementary material for: Integration Analysis of JAK2 or RUNX1 Mutation With Bone Marrow Blast Can Improve Risk Stratification in the Patients With Lower Risk Myelodysplastic Syndrome
Source: Front Oncol. 2021 Jan 13;10:610525. doi: 10.3389/fonc.2020.610525 (PMC7839382; doi:10.3389/fonc.2020.610525)
Supplement: Supplementary Table 1 — Genes for targeted sequencing. [file Table_1.docx]

**Table S1. Genes for targeted sequencing**

| 29 gene panel | | | | | |
| --- | --- | --- | --- | --- | --- |
| ANKRD11 | DHX9 | IDH2 | PTPRD | SF3B1 | U2AF1 |
| ASXL1 | DNMT3A | ITIH3 | ROBO1 | SRSF2 | UPF3A |
| BCOR | EZH2 | JAK2 | ROBO2 | STAG2 | WT1 |
| CALR | GATA2 | KIF20B | RUNX1 | TET2 | ZRSR2 |
| CEBPA | IDH1 | MPL | SETBP1 | TP53 |  |
