# Supplementary figures and images for: Integration Analysis of JAK2 or RUNX1 Mutation With Bone Marrow Blast Can Improve Risk Stratification in the Patients With Lower Risk Myelodysplastic Syndrome
Source: Front Oncol. 2021 Jan 13;10:610525. doi: 10.3389/fonc.2020.610525 (PMC7839382; doi:10.3389/fonc.2020.610525)

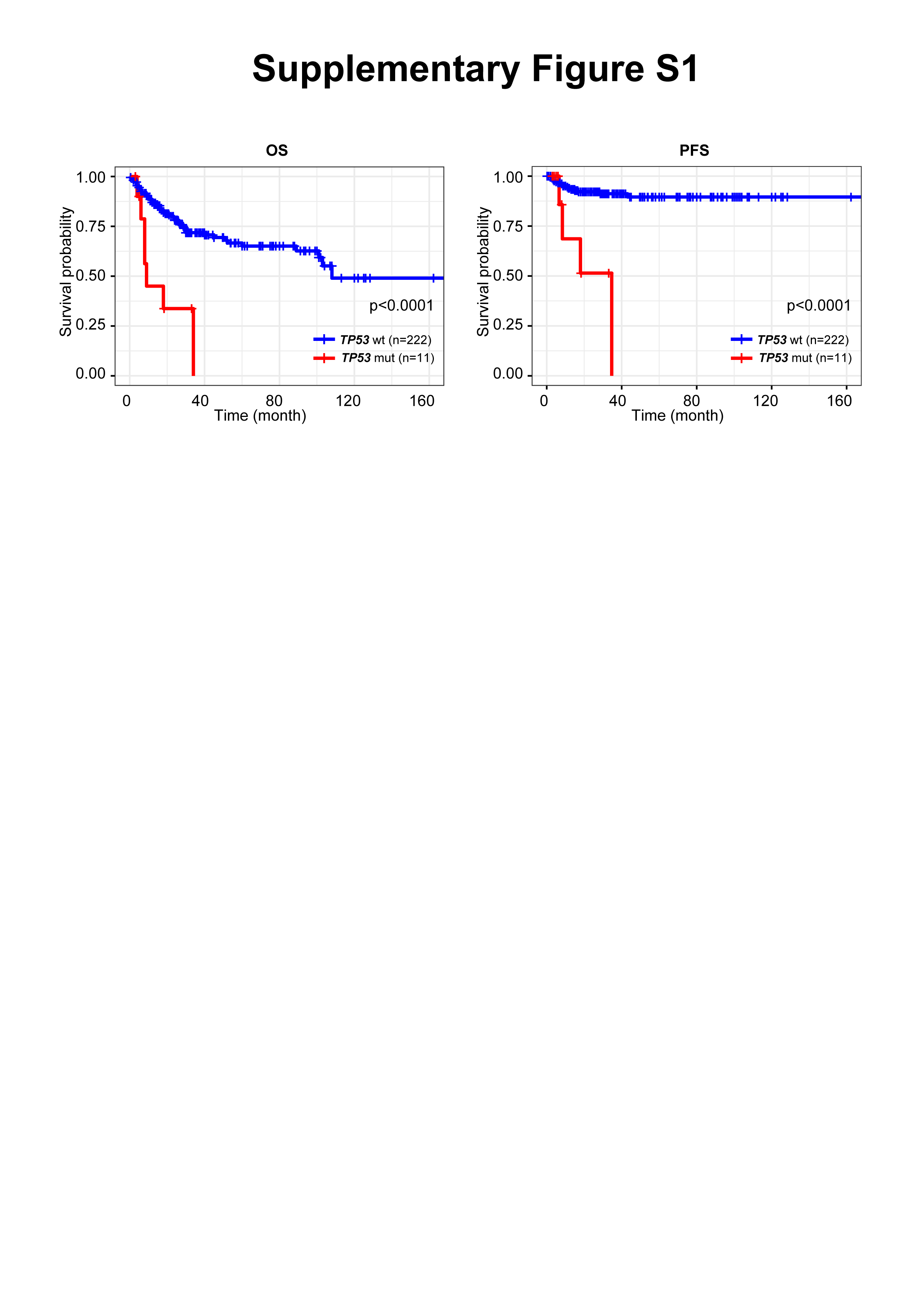

Supplement: Supplementary Figure 1 — Kaplan-Meier curves for OS and PFS according to TP53 status. [file Image_1.png]
